# RECQ4 restricts non‐interfering crossover formation to fine‐tune meiotic recombination rates in rice

**DOI:** 10.1111/pbi.70181

**Published:** 2025-06-15

**Authors:** Yafei Li, Hanli You, Xinjie Cheng, Bingxin Wang, Yangzi Zhao, Jiawei Chen, Lei Cao, Minsi Wen, Yansong Zhang, Zhuoran Chen, Qiong Luo, Zhukuan Cheng

**Affiliations:** ^1^ State Key Laboratory of Plant Genomics, Institute of Genetics and Developmental Biology, Innovation Academy for Seed Design Chinese Academy of Sciences Beijing China; ^2^ Jiangsu Key Laboratory of Crop Genomics and Molecular Breeding/Key Laboratory of Plant Functional Genomics of the Ministry of Education, Jiangsu Co‐Innovation Center for Modern Production Technology of Grain Crops Yangzhou University Yangzhou China; ^3^ Ministry of Education Key Laboratory of Agriculture Biodiversity for Plant Disease Management Yunnan Agricultural University Kunming China; ^4^ College of Advanced Agricultural Sciences University of Chinese Academy of Sciences Beijing China

**Keywords:** rice, meiosis, RECQ4, FANCM, recombination frequency, crop improvement

## Abstract

Crop breeding fundamentally depends on meiotic crossovers (COs) to reshuffle genetic material and integrate favourable alleles into elite cultivars. Recombination frequency is of paramount importance in this process. Higher recombination rates enhance the probability of breaking linkage drag and generating novel allelic combinations. Here, using rice (*Oryza sativa*) as a model crop species, we reveal that RECQ4, a conserved suppressor of meiotic CO formation, is indispensable for safeguarding the integrity of meiotic recombination intermediate metabolism. We demonstrate that RECQ4 limits COs in rice by specifically suppressing non‐interfering CO pathways. Genetic redundancy with FANCM underscores their cooperative function in ensuring canonical CO formation, which is essential for accurate homologue segregation and genome stability. Furthermore, the *recq4 dmc1* double mutant exhibits persistent chromosome fragmentation, implicating RECQ4 in resolving recombination intermediates through sister chromatid repair. Our findings redefine RECQ4's role in crop meiosis, bridging its CO suppression activity with broader genome surveillance functions.

## Introduction

Meiotic recombination is a fundamental process in sexual reproduction. First, the formation of crossovers (COs) is essential for accurate chromosome segregation during gamete formation. Second, the reciprocal exchange of genetic material between homologous chromosomes facilitates the reshuffling of parental genetic information, ensuring that recombined material is passed on to the next generation. This makes meiotic CO formation a key target in crop breeding. Meiosis begins with the introduction of numerous DNA double‐strand breaks (DSBs), most of which are repaired through non‐CO events and only a few of them lead to the formation of recombinant chromosomes.

Meiotic recombination is initiated by programmed DSBs, which are catalysed by the SPO11 and MTOPVIB proteins (Keeney *et al*., 1997; Robert *et al*., 2016; Xue *et al*., 2016). These DSBs are subsequently processed by strand resection, forming 3′‐ended single‐stranded DNA (ssDNA) tails. These ssDNA tails are immediately bound by RPA proteins, which are subsequently replaced by DMC1 or RAD51 to invade homologous sequences.

Both RAD51 and DMC1 recombinases catalyse meiotic break repair, ensuring COs occur between homologous chromosomes (inter‐homologue, IH) rather than between adjacent sister chromatids (inter‐sister, IS). Recent research, primarily focused on budding yeast meiosis, suggests that meiotic break repair occurs in two distinct temporal phases: an initial DMC1‐permissive phase (Phase 1), followed by a RAD51‐permissive phase (Phase 2) (Ziesel *et al*., 2022). During the DMC1‐permissive phase, DMC1 primarily repairs DSBs and facilitates inter‐homologue recombination, while RAD51 remains catalytically inactive (Tsubouchi & Roeder, 2006; Busygina *et al*., 2008; Niu *et al*., 2009; Lao *et al*., 2013; Callender *et al*., 2016). In the RAD51‐permissive phase, the RAD51‐mediated pathway becomes active, repairing the remaining DSBs, predominantly using sister chromatids (Crismani *et al*., 2013; Enguita‐Marruedo *et al*., 2019; Toraason *et al*., 2021; Emmenecker *et al*., 2024). However, in plants, RAD51 can repair breaks in the absence of DMC1, although it does so by using sister chromatids, as evidenced by the near absence of inter‐homologue COs (Couteau *et al*., 1999; Wang *et al*., 2016).

Two distinct types of COs are known. Class I CO formation depends on a set of meiosis‐specific proteins known as the ZMMs (ZIP1, ZIP2, ZIP3, ZIP4, MER3, MSH4, PTD, HEIP1 and MSH5) (Li *et al*., 2018; Li *et al*., 2023; Luo *et al*., 2013; Shen *et al*., 2012; Wang *et al*., 2009; Wang *et al*., 2010; Wang *et al*., 2012; Zhang *et al*., 2014). These COs exhibit interference, meaning they are spaced widely along chromosomes. In organisms like budding yeast and *Arabidopsis*, Class I COs account for 80%–90% of total COs (Lambing *et al*., 2017; Osman *et al*., 2011). The remaining COs, classified as Class II, are partially dependent on the MUS81 endonuclease (Higgins *et al*., 2008; Mu *et al*., 2023; Osman *et al*., 2011).

In *Arabidopsis*, extensive research by Mercier and colleagues has identified several factors that inhibit CO [anti‐crossover (anti‐CO)] during meiosis, particularly those limiting the formation of Class II COs (Crismani *et al*., 2012; Fernandes *et al*., 2018a, 2018b; Girard *et al*., 2014; Séguéla‐Arnaud *et al*., 2015, 2017; Serra *et al*., 2018). These anti‐CO factors are grouped into three main categories: the FANCM helicase and its cofactors (MHF1 and MHF2), the BLM‐TOP3α–RMI complex and the FIGNL1‐FLIP complex. These factors have additive effects on CO formation but have little to no impact on fertility. Subsequent studies in various plants (Arrieta *et al*., 2021; Blary *et al*., 2018; Desjardins *et al*., 2022; Li *et al*., 2021, 2023; Mieulet *et al*., 2018; Osman *et al*., 2024), including crops like wheat, rice, tomato and pea, have confirmed the conserved nature of these gene functions, while also revealing some species‐specific differences, suggesting potential divergence in their roles. Additionally, the loss of rice RPA1a function results in a significant release of repression on Class II COs, although this is accompanied by sterility (Miao *et al*., 2021).

Increasing CO rates can enhance the combination of beneficial traits from different parent lines, thus improving breeding efficiency. Rice, a staple crop that feeds more than half of the world's population, is vital for addressing challenges related to population growth and environmental pressures, such as climate change. Developing improved rice varieties is crucial to ensure food security. Although increased CO rates have been documented in the rice *RECQ4* mutant (Mieulet *et al*., 2018), the molecular mechanisms underlying this phenotype remain poorly understood. Crucially, the functional interaction between RECQ4 and FANCM helicase, particularly their coordinated regulation of Class II CO formation, has yet to be characterized in this species.

## Results

### RECQ4 is dispensable for rice fertility

**Figure 1:**
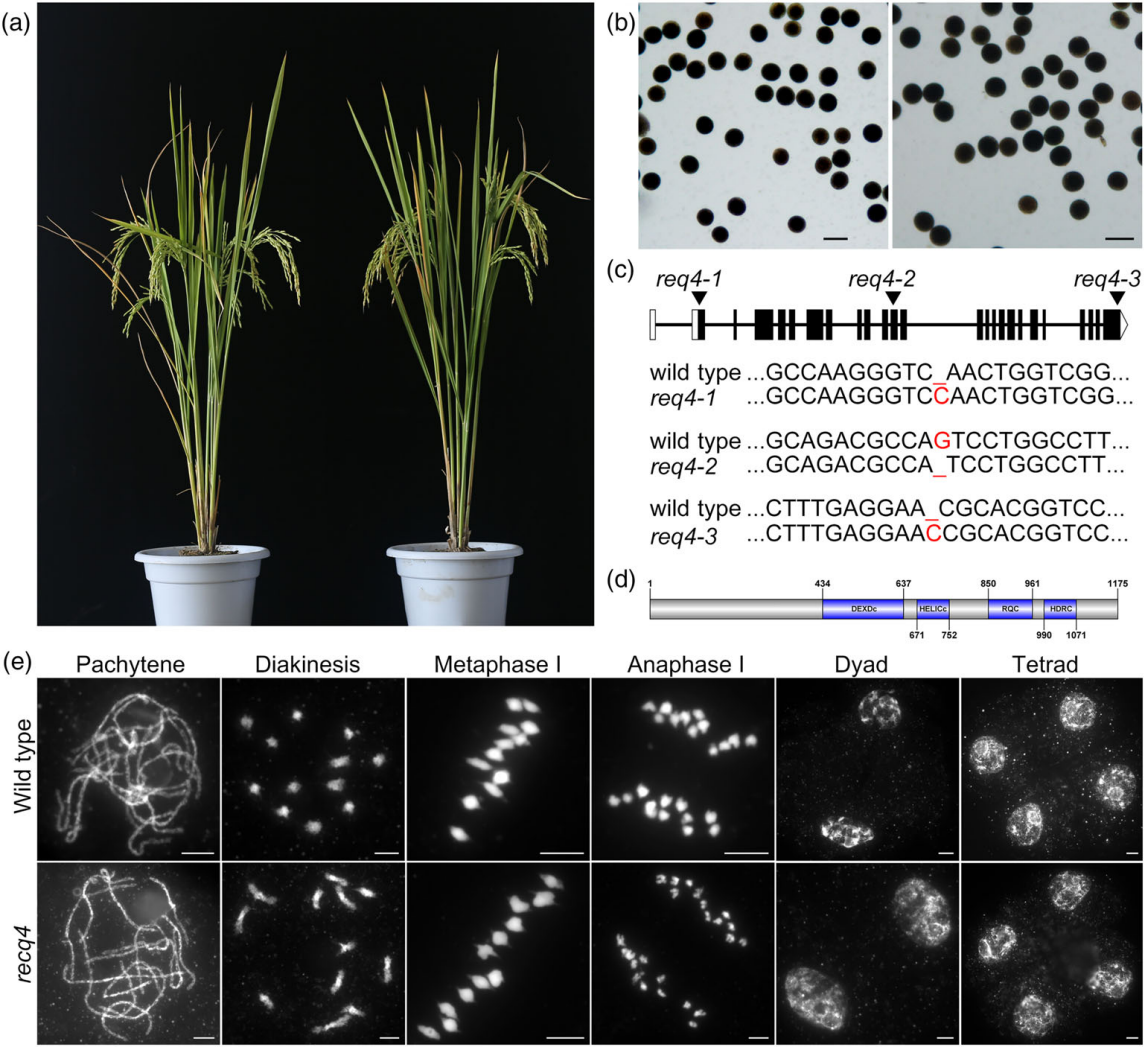
Characterization of RECQ4. (a) RECQ4 is not required for fertility in rice: comparison of wild type (left) and the *recq4‐1* mutant (right) plants. (b) Pollen grains stained with 1% I_2_‐KI solution from the wild‐type plant (left) and the *recq4‐1* mutant (right). (c) Gene structure of RECQ4 and the mutated sites. Coding sequences (CDS) are represented by black‐filled boxes and untranslated regions (UTRs) by white‐filled boxes. (d) Schematic showing the domains in the RECQ4 protein. Grey boxes represent non‐domain regions, while blue boxes indicate four distinct domains. (e) Meiotic chromosome behaviours in the wild type and the *recq4‐1* mutant. All bars = 5 μm.

To further investigate the meiotic function of RECQ4, we utilized the clustered regularly interspaced short palindromic repeats (CRISPR)/CRISPR‐associated protein 9 (Cas9) system to generate specific mutations in this gene. Three independent transgenic lines were obtained in the rice variety Zhongxian 3037 (Figure 1). All three mutants exhibited normal vegetative growth and pollen viability compared with the wild type (Figure 1a,b). Among these, we selected *recq4‐1* for further analysis (Figure 1c,d). In the wild type, chromosomes underwent condensation to form long, thin threads during leptotene. Through zygotene and pachytene, homologous chromosomes paired and synapsed. From diplotene to diakinesis, after synaptonemal complexes disassembled, COs between homologous chromosomes matured into visible chiasmata, chromosomes further condensed and 12 bivalents were distinctly observed. The bivalents aligned in an orderly manner on the equatorial plate during metaphase I, followed by the separation and migration of homologous chromosomes in opposite directions during anaphase I. In the second meiotic division, sister chromatids of each chromosome separated, yielding four daughter cells, each containing 12 chromosomes (Figure 1e). Male meiosis in the *recq4* mutant showed no detectable defects, consistent with wild‐type fertility.

### RECQ4 mutations increase bivalent formation in rice *zmm* mutants

**Figure 2:**
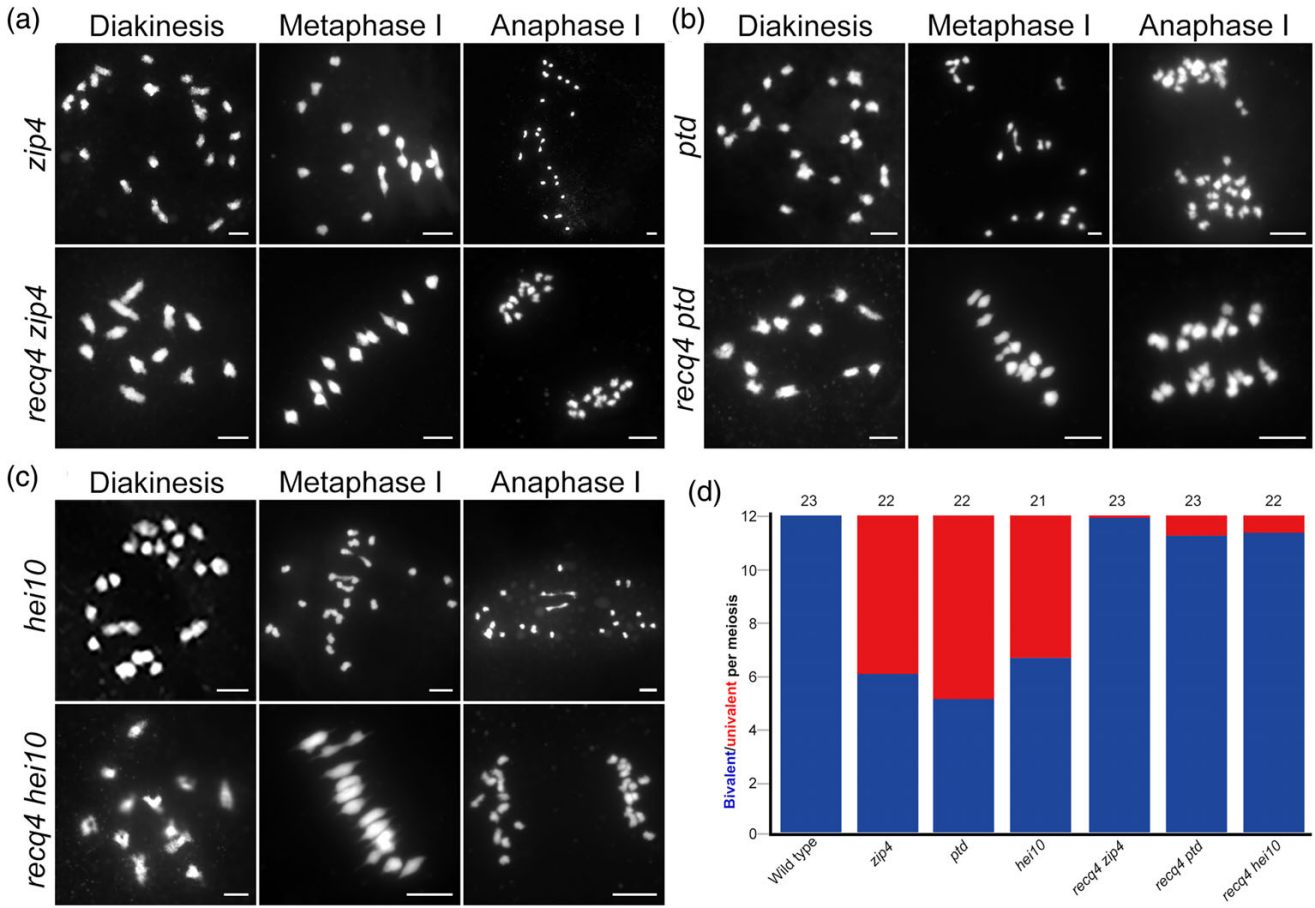
Loss of RECQ4 nearly completely restores bivalent formation in ZMM mutants. (a–c) Genetic analysis of RECQ4 with ZIP4, PTD and HEI10. The chromosomal behaviour in the *recq4 zip4*, *recq4 ptd* and *recq4 hei10* double mutants closely resembles that of the *recq4* single mutant. ZIP4, PTD and HEI10 are typical ZMM proteins, and mutations in these ZMM proteins result in a univalent phenotype at diakinesis. All bars = 5 μm. (d) Average number of bivalents per male meiocyte in the *zip4*, *recq4 zip4*, *ptd*, *recq4 ptd*, *hei10* and *recq4 hei10* single and double mutants. Blue and red bars represent pairs of univalents and bivalents, respectively. The number of cells analysed is shown above each bar.

The meiotic anti‐CO activity of *RECQ4* was initially characterized via a genetic screen, as its mutation restores bivalent formation in *ZMM* mutants in *Arabidopsis* (Arrieta *et al*., 2021). We further investigated the relationship between *ZMM* and *RECQ4* in monocots, specifically in rice. The *recq4 zmm* double mutants were generated, and we quantified the number of bivalents alongside the *hei10*, *ptd* and *zip4* mutants. This was compared with the bivalent numbers in *recq4 hei10*, *recq4 ptd* and *recq4 zip4* mutants by studying chromosome configurations at metaphase I. The *recq4 zmm* double mutants exhibited a significantly increased bivalent frequency compared with the *ZMM* mutants alone with the corresponding genotype. In the *recq4 zmm* double mutants, 12 bivalents were readily detected, which were not observed in the *ZMM* mutants, suggesting that RECQ4 restricts meiotic CO formation (Figure 2).

### DSB formation, ssDNA‐DMC1 binding and SC assembly appear normal in *recq4*

**Figure 3:**
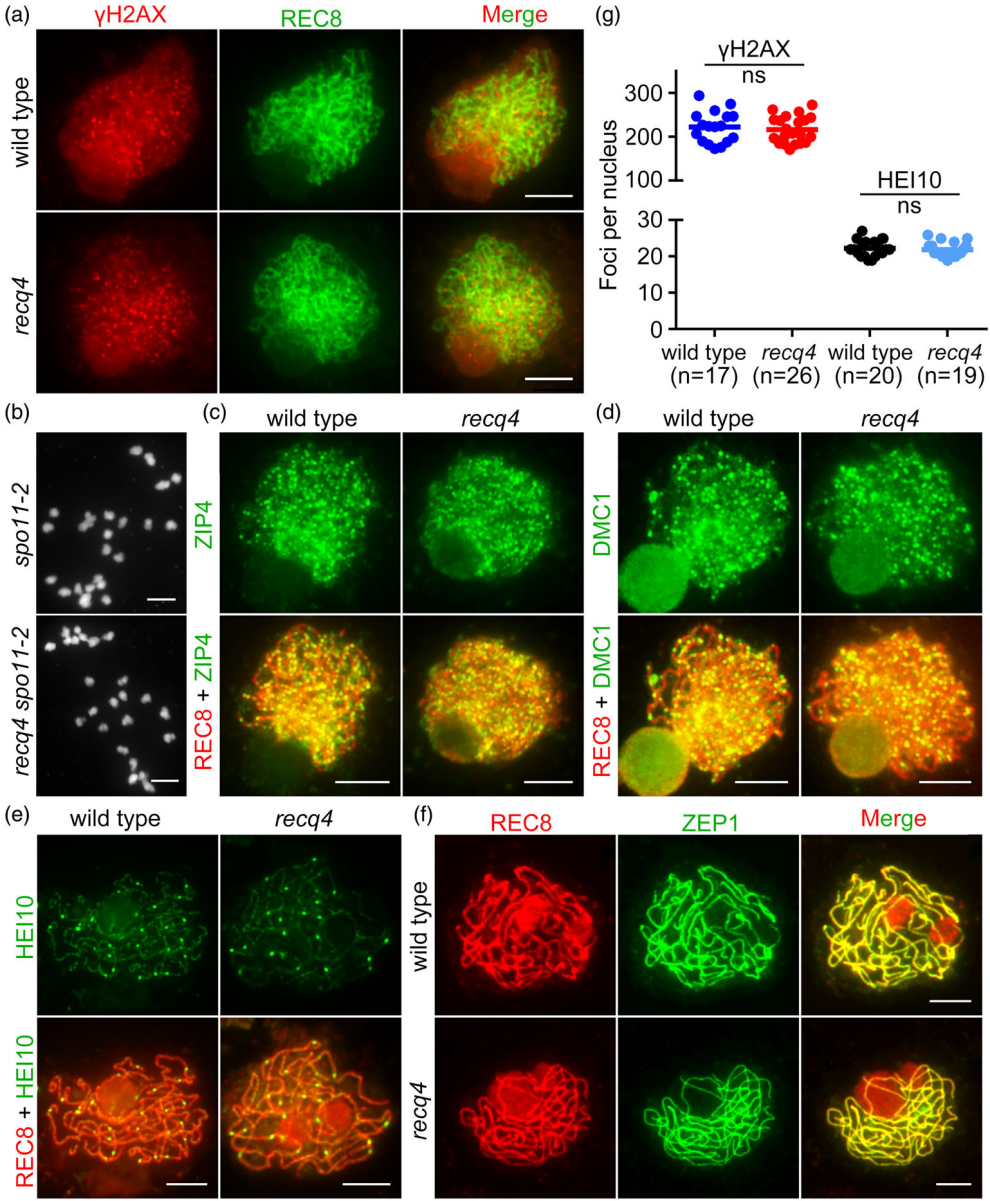
Meiotic recombination events are normal in the *recq4* mutant. (a) Immunolocalization of histone H2AX phosphorylation (γH2AX) in wild type (WT) and *recq4* mutants. REC8 (green) is used to determine meiotic stages, while γH2AX (red) serves as a reliable marker for double‐strand breaks (DSBs). (b) Genetic analysis of RECQ4 with SPO11‐2. Mutation of SPO11‐2 results in 24 univalents at diakinesis. (c) and (d) Immunolocalization of ZIP4 (green) and DMC1 (green) in WT and *recq4* mutants. REC8 (red) is used to determine meiotic stages. (e) Immunolocalization of HEI10 (green) in WT and the *recq4* mutants. REC8 (red) is used to determine meiotic stages. The number of bright HEI10 foci indicates the number of Class I crossovers (COs). (f) Immunostaining for the transverse filament protein of the synaptonemal complex (ZEP1) in WT and *recq4* mutants at pachytene. ZEP1 (green) serves as a reliable marker for synapsis, while REC8 (red) is used to determine meiotic stages. (g) Quantification of γH2AX and Hei10 foci in WT and *recq4* mutants. Statistical analysis showed no significant differences (ns) between WT and *recq4*. All bars = 5 μm.

To determine whether RECQ4 has an impact on the number of DSBs, we performed an immunolocalization study with anti‐γH2AX in *recq4* meiocytes. The mean number of γH2AX foci was 217 ± 30 (*n* = 26) in *recq4* and 223 ± 36 (*n* = 17) in wild type at the same stage and did not differ significantly (unpaired *t*‐test, *P* = 0.60), suggesting that DSB formation occurs normally in *recq4* (Figure 3a). To further confirm this, we generated the *recq4 spo11‐2* double mutants (Figure S1), which appeared similar to the *spo11‐2* single mutant phenotype with 24 randomly scattered univalents during the first meiotic division (Figure 3b). Therefore, RECQ4 functions in response to meiotic DSBs.

Furthermore, we performed immunostaining using the antibodies against DMC1, the meiosis‐specific recombinase, which mediates inter‐homologue strand exchange (Figure 3d; Figure S2). The number of DMC1 foci did not significantly differ (unpaired *t*‐test, *P* = 0.05) in the *recq4* mutant (239 ± 9, *n* = 24) from that in wild type (264 ± 9, *n* = 26) at zygotene, suggesting that the CO increase is not associated with increased DSBs.

Based on 4′,6‐diamidino‐2‐phenylindole (DAPI) staining, the synapsis of homologous chromosomes was essentially normal in *recq4* meiocytes (Figure 1e). In order to confirm the SC formation in *recq4* mutants, we performed immunolocalization with antibodies against ZEP1, the transverse filament protein of SC. The localization of ZEP1 was indistinguishable from that of wild type at pachytene (Figure 3f), suggesting that SC formation was completed in *recq4*.

### *recq4* exhibits a normal number of bright HEI10 foci

The interference‐sensitive pathway (CO I), which depends on ZMM protein (such as ZIP4), accounts for most of the COs in rice. In order to verify the frequency of interference‐sensitive CO formation in *recq4*, we first determined the localization of ZIP4 in both wild type and *recq4* mutant plants. The distributions of ZIP4 in *recq4* were similar to those in wild type (Figure 3c), suggesting that ZMM‐dependent COs formed normally. Next, we found that the mean number of HEI10 bright foci of *recq4* (21 ± 2, *n* = 19) did not differ significantly (unpaired *t*‐test, *P* = 0.55) compared with that of the wild type (22 ± 2, *n* = 20). Therefore, the formation of interference‐sensitive COs proceeds normally in *recq4* during rice meiosis (Figure 3e,g).

### MUS81 is required for resolving recombination intermediates in *recq4*

**Figure 4:**
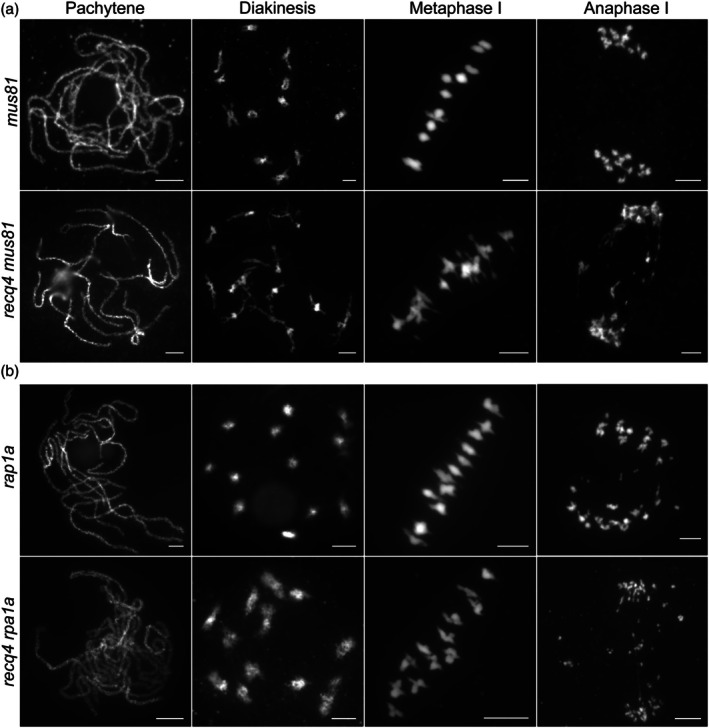
Genetic analysis of RECQ4 with MUS81 and RPA1a. (a) Chromosome behaviours in *recq4 mus81* double mutant at different meiotic stages. (b) Chromosome behaviours in *recq4 rpa1a* double mutant at different meiotic stages. All bars = 5 μm.

The lack of viability of the *recq4 mus81* double mutant in *Arabidopsis* restricts further research on meiosis involving this genotype in previously reported (Arrieta *et al*., 2021). Fortunately, we obtained *recq4 mus81* mutants in rice. Despite exhibiting growth defects characterized by lower plant height (Figure S3), meiocytes could be obtained from *recq4 mus81* plants. Meiotic progression from leptotene to metaphase I appeared normal, as evidenced by DAPI staining of meiotic chromosomes in PMCs (Figure 4a). The detection of chromosome fragmentation during anaphase I corresponds to the observed sterility, a phenomenon absent in *recq4* or *mus81* single mutants (Mu *et al*., 2023). This observation suggests that MUS81 becomes indispensable for the comprehensive repair of recombination intermediates in *recq4*.

### Chromosome behaviour in *rpa1a recq4* double mutants recapitulates the *rpa1a* single mutant phenotype

Our previous studies have shown that the scaffold protein RPA1a plays a crucial role in limiting redundant chiasma formation during rice meiosis (Miao *et al*., 2021). This function is partially attributed to the abnormal activity of the Bloom syndrome protein (BLM) homologue, RECQ4, in the BLM‐TOP3α–RMI complex. Guided by these findings, we suspected a mutation in RECQ4, a member of this complex, in the *rpa1a* background would not lead to changes in chromosome behaviour compared with the *rpa1a* single mutant. To find the difference in chromosome morphology among the two single mutants, *recq4* and *rpa1a*, and the double mutant *recq4 rpa1a*, we carefully investigated their chromosome behaviours during anaphase I. The *recq4* was close to the wild type without any chromosome fragments at anaphase I, while *rpa1a* was similar to the double mutants, all with chromosome fragments at anaphase I (Figure 4b; Figure S1). Thus, we hypothesized that both RPA1A and RECQ4 function to restrict non‐interfering CO formation, but RPA1A likely acts upstream of RECQ4 in this regulatory pathway.

### Functional overlap between RECQ4 and FANCM ensures accurate meiotic chromosome segregation during rice meiosis

The CO frequency was increased by ninefold in *Arabidopsis recq4 fancm* double mutants (Séguéla‐Arnaud *et al*., 2015), which encourages us to explore the meiotic function of the *recq4 fancm* double mutants in crop species. We generated the double mutant in rice using two methods: CRISPR technology and hybridization. The *recq4 fancm* double mutant displays normal vegetative growth but is nearly sterile (Figure S4). To investigate the underlying cause of sterility in *recq4 fancm* mutants, we analysed the meiotic chromosomes of pollen mother cells at various meiotic stages in both the wild type and the *recq4 fancm* mutant.

**Figure 5:**
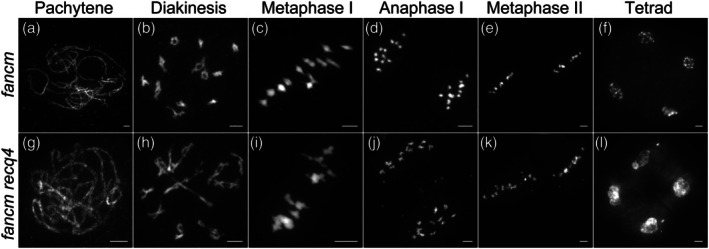
Genetic analysis of RECQ4 with FANCM. (a–f) Chromosome behaviour in *fancm* mutant at different meiotic stages. (g–l) Chromosome behaviour in *fancm recq4* double mutant at different meiotic stages. All bars = 5 μm.

The chromosomal behaviour of *recq4 fancm* double mutants was indistinguishable from the wild type from leptotene to pachytene. However, anomalies began to manifest thereafter (Figure 5). Unlike the 12 intact condensing bivalents observed in wild type or *fancm* mutants (Figure 5b), these abnormal chromosome interactions became more pronounced at diakinesis or metaphase I (Figure 5 h,i), where chromosomes appeared as entangled masses, although bivalent‐like structures still existed (Figure 5i). During anaphase I, extensive chromosome bridges and fragments were observed on the plate (Figure 5g). Following meiosis II (Figure 5k), tetrads with unequal chromosome distribution and micronuclei formed (Figure 5l). Similar defects were observed during meiosis in the T‐DNA insertion double mutant of *recq4 fancm* (Figure S5). These data underscore the genetic redundancy between RECQ4 and FANCM, which safeguards accurate meiotic chromosome segregation.

### Loss‐of‐function in both RECQ4 and FANCM exhibits a greater capacity to rescue bivalent formation in ZMM‐deficient backgrounds

**Figure 6:**
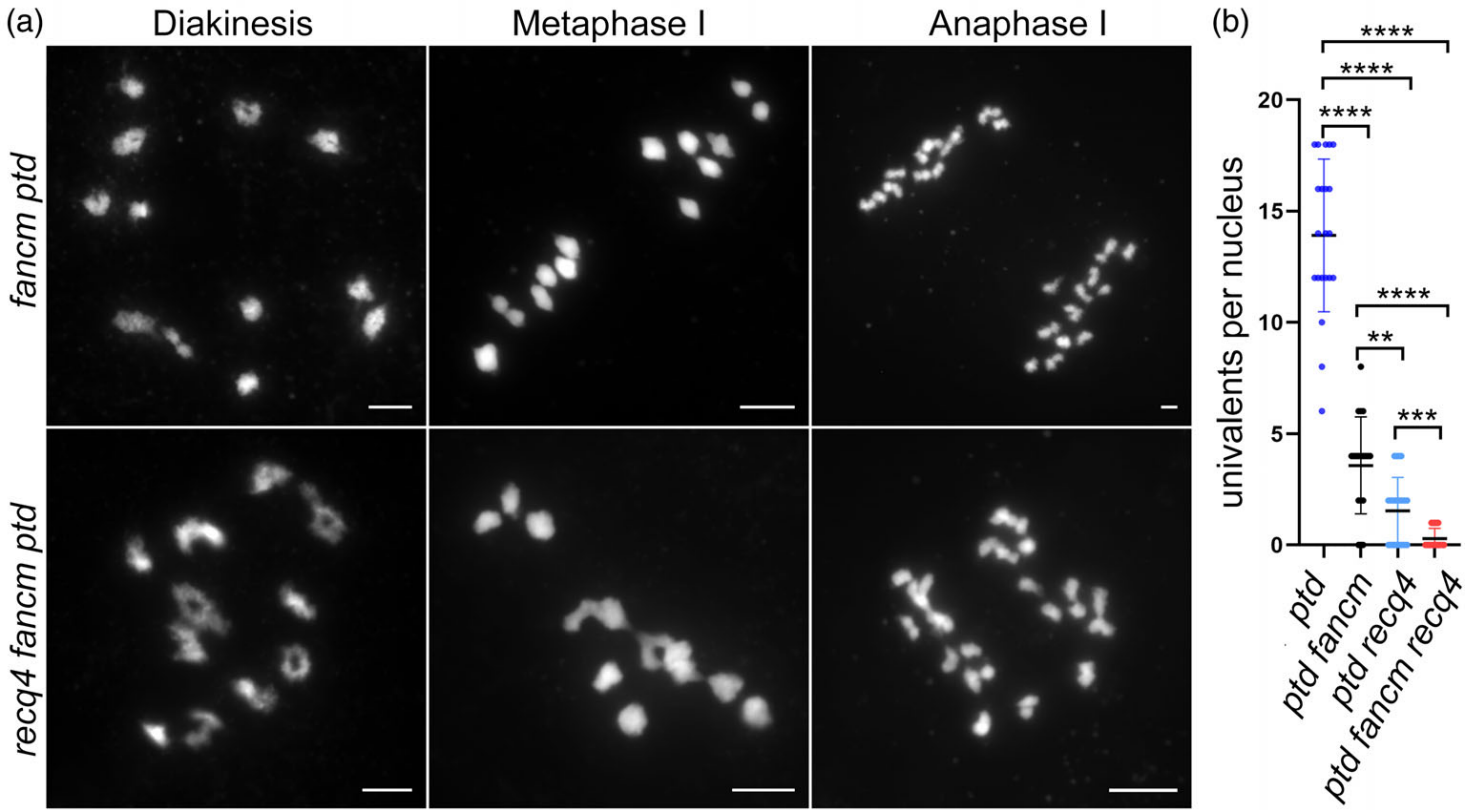
Increased number of crossovers is present in the *recq4 fancm* double mutants. (a) Chromosome behaviour in double or triple mutants at different meiotic stages. All bars = 5 μm. (b) Scatter plots display the number of univalents in various mutants.

We generated *recq4 fancm ptd* triple mutants (TMs) to examine whether univalent formation was further reduced compared with the *recq4 ptd* double mutant using cytological assessments (Figure 6; Figure S1). We observed a significant decrease (unpaired *t*‐test, *P* < 0.01) in univalent formation in the *recq4 fancm ptd* TM (0.29 ± 0.09 per cell, *n* = 24), compared with both *recq4 ptd* (1.55 ± 0.32 per cell, *n* = 22) and *fancm ptd* double mutants (3.58 ± 0.50 per cell, *n* = 19) (Figure 6b). As a result, we suspected that the loss‐of‐function mutation in both RECQ4 and FANCM may exhibit a greater capacity to rescue bivalent formation in ZMM‐deficient backgrounds.

### RECQ4 may be involved in the repair of meiotic DSBs via sister chromatids

**Figure 7:**
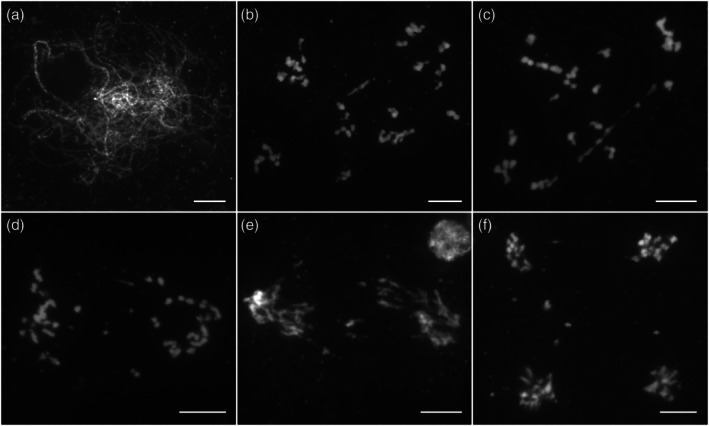
Meiotic chromosomes stained with 4',6‐Diamidino‐2‐Phenylindole in the *recq4 dmc1* double mutant pollen mother cells. (a) Pachytene. (b) Diakinesis. (c) Metaphase I. (d) Anaphase I. (e) Telophase I. (f) Telophase II. All bars = 5 μm.

The role of RECQ4 in the context of DMC1 deficiency has not been extensively studied. To investigate its function, we generated a *recq4 dmc1* double mutant. DAPI staining analysis of the double mutant revealed abnormal synapsis of homologous chromosomes, although some regions of proper alignment remained visible (Figure 7a). As condensation progressed, a significant number of irregularly shaped univalents were observed at the diakinesis stage (Figure 7b). These univalents failed to align properly along the equatorial plate and remained scattered throughout the nucleus (Figure 7c). During anaphase I, all chromosomes segregated to the poles, with chromosome fragments detected (Figure 7d). Chromosome fragments were consistently observed during subsequent telophase I (Figure 7e) and tetrad stages (Figure 7f). Based on these findings, we cautiously conclude that RECQ4 is essential for the repair of DSBs through sister chromatids.

## Discussion

### Differentiation of RECQ4 function across species

*Arabidopsis* RECQ4A and RECQ4B originated from a recent chromosomal segment duplication within the *Brassicaceae* family, leading to the formation of two genes with highly similar protein sequences. The *recq4a recq4b* double mutant was able to restore both bivalent formation and fertility in a *zmm* background. Despite a 6.2‐fold increase in genetic distances, elevated recombination did not compromise chromosome segregation or fertility in *recq4a recq4b* mutants (Séguéla‐Arnaud *et al*., 2015). Barley, tomato and rice each have only one homologous gene. The *HvRECQL4* mutant resulted in the most complete restoration of fertility in the semi‐fertile barley mutant *mlh3*. Recombination levels in homozygous *HvRECQL4* lines were nearly double those in the wild type of barley. de Maagd *et al*. first demonstrated in tomato that *recq4* increases recombination frequency in interspecific hybrids (de Maagd *et al*., 2020). A biallelic *recq4* mutant was created in the F1 hybrid of *Solanum lycopersicum* and *S. pimpinellifolium*. Compared with the wild‐type F1 hybrid, the *recq4* mutant showed a significant increase in COs: a 1.53‐fold increase in ring bivalents observed microscopically in male meiocytes, and a 1.8‐fold extension of the genetic map based on SNP marker analysis in the F2 progeny. Through cytological analyses, our study independently confirms that RECQ4 limits meiotic CO in rice. In a wheat (*Triticum aestivum* L.) TM lacking the three homoeologous copies of TaRECQ4, phenotypic observations revealed a significant reduction in fertility and pollen viability. The presence of numerous multivalents in TM plants suggests that TaRECQ4 may also play a role in regulating homoeologous recombination (Bazile *et al*., 2023). Building on the above results, the role of the *RECQ4* in allopolyploids deserves further exploration.

### How high can recombination rate go during rice meiosis?

*Arabidopsis*, the model dicot plant with the smallest genome, showed the greatest effect when combining *recq4* and *figl1* mutations. This resulted in a significant increase in hybrid genetic map length, from 389 to 3037 cM, corresponding to an unprecedented 7.8‐fold increase in CO frequency (Fernandes *et al*., 2018a). Disrupting the three pathways further did not lead to any additional increase in recombination, suggesting that an upper limit had been reached.

Here, we have conducted several anti‐CO tests in rice (Hu *et al*., 2017; Li *et al*., 2023; Yang *et al*., 2022). Given the functional redundancy between RECQ4 and FANCM, our findings support the conclusion that manipulating RECQ4 could serve as a universal tool for enhancing recombination in plants (Mieulet *et al*., 2018), with the exception of allopolyploid wheat (Bazile *et al*., 2023). However, it is important to note that data obtained from model species may not always be directly applicable to crops.

We speculate that the significant difference between the conclusions of this study and those in *Arabidopsis* may stem from the considerably larger genome size of rice, which appears to be associated with a higher frequency of DNA DSB per cell. In *Arabidopsis recq4 fancm* double mutants, the DNA repair machinery can effectively manage a relatively low number of aberrant recombination intermediates, resulting in more COs and fewer unrepaired DNA molecules. However, in rice, the increased number of aberrant recombination intermediates necessitates that at least one of FANCM or RECQ4 remains functional.

### Don't forget your sister: Involvement in DSBs repair during rice meiosis

One of the hallmark features of meiosis is the formation of COs between homologous chromosomes. The meiotic double‐strand break repair (DSBR) model predominantly highlights IH repair (An *et al*., 2003), yet sister chromatid repair (IS), which ensures genomic fidelity via homologous recombination between sister chromatids, has not been adequately investigated (Humphryes and Hochwagen, 2014).

The meiotic template choice (IH vs. IS) relies on precise regulation of RAD51 and DMC1 recombinases to ensure genetic diversity and fidelity. In *recq4 dmc1* double mutants, extensive chromosome fragmentation was observed in all cells from anaphase I through the remainder of meiosis, a phenotype absent in the *dmc1* single mutant. This difference implies that RECQ4 may also function in meiotic DSBR via a sister chromatid‐dependent pathway.

## Materials and methods

### Plant material

The meiotic mutants used in this study, *zip4* (Shen *et al*., 2012), *ptd* (Li *et al*., 2023), *hei10* (Wang *et al*., 2012), *mus81* (Mu *et al*., 2023), *spo11‐2* (Miao *et al*., 2021), *dmc1* (Wang *et al*., 2016) and *rpa1a* (Miao *et al*., 2021), were previously isolated in our laboratory. Double mutants combining these with *recq4* were generated through crosses. The *fancm*, *recq4 fancm* double mutants (Figure S1) were generated in the rice variety Zhongxian 3037 through CRISPR/Cas9‐mediated mutagenesis (Wang *et al*., 2015). The T‐DNA mutants of FANCM (5A‐00166) and RECQ4 (3A‐01037), also used to construct the double mutants, were sourced from the POSTECH Rice Insertion Database (An *et al*., 2003). The *recq4 fancm ptd* TMs were generated by introducing mutations into the RECQ4 and FANCM genes using CRISPR/Cas9 gene editing in the progeny of self‐fertilized PTD heterozygous plants. These heterozygous plants were derived from the rice variety Yandao 8. The genotype of the TMs (*recq4 fancm ptd*) was confirmed through PCR sequencing. All plants were grown in experimental fields of Beijing or Hainan under normal growth conditions.

### Meiotic chromosome preparation

Carnoy's solution (ethanol and glacial acetic acid at a 3:1 ratio) was used to fix young panicles (6 ± 1 cm) from both wild‐type and mutant plants. Microsporocytes from the young panicles were selected, and the anthers were squashed in acetocarmine solution under a cover slip. After freezing in liquid nitrogen, the coverslips were quickly removed (within 1 min), and the slides containing chromosomes were dehydrated through a series of alcohol washes. The alcohol series consisted of absolute ethanol and sterile deionized water in the following concentrations: 70%, 90% and 100% ethanol (Cheng, 2013). Chromosomes on the slides were stained with DAPI in an antifade solution (Vector Laboratories, Burlingame, CA), and the chromosomes were observed and imaged using a ZEISS A2 fluorescence microscope equipped with a micro‐CCD camera.

### Immunofluorescence

A 4% (w/v) paraformaldehyde solution was used to fix young panicles from both wild type and mutant plants. After fixation at room temperature (20–25 °C) for 30 min, the anthers were squashed with a dissecting needle in PBS solution and covered with a coverslip. The slides containing chromosomes were then frozen in liquid nitrogen. After removing the coverslips, the slides were dehydrated through a series of ethanol washes (70%, 90% and 100%) (Cheng, 2013). The slides were subsequently incubated in a humid chamber with different antibody combinations, each diluted 1:500 in 1× TNB buffer (0.1 M Tris–HCl, pH 7.5, 0.15 M NaCl and 0.5% blocking reagent) at 37 °C for 4 h. Following three rounds of PBS washes, Texas‐Red‐conjugated goat anti‐rabbit antibody and fluorescein isothiocyanate (FITC)‐conjugated sheep anti‐mouse antibody (1:1000) were applied to the slides. Chromosomes were counterstained with DAPI in an antifade solution (Vector Laboratories). All images were captured using a Carl Zeiss Axio Imager A2 microscope equipped with a micro‐CCD camera.

### CRISPR‐Cas9 targeting of *RECQ4* and *FANCM*

The target sequences were GACTTCGACTGGGAGGCGG and TGATAAAGCCAAGGGTCAACTGG, respectively. The CRISPR‐Cas9 binary vector pC1300‐cas9 was employed for genetic transformation.

### Statistical analysis

Statistical significance was determined by an unpaired two‐tailed *t*‐test, unless otherwise stated. GraphPad Prism 6 (http://www.graphpad.com/) was used to produce graphs.

## Author contributions

YFL performed the experiments, analysed the data and wrote the paper. HLY, XJC, BXW, YZZ and JWC participated in performing experiments. YSZ and ZRC participated in material production. LC and MSW participated in data analysis. QL and ZKC supervised and completed the writing.

## Conflicts of interest

The authors declare that they have no conflict of interest.

## Supporting information


**Figure S1** Characterization of the mutants.
**Figure S2** Quantification of DMC1 foci in wild type and the *recq4* mutant.
**Figure S3** Phenotypic comparison between the *mus81* mutant and the *mus81 recq4* double mutant.
**Figure S4** Phenotypic comparison between wild type and the *recq4 fancm* double mutant.
**Figure S5** Meiotic chromosome behaviours and pollen viability in the T‐DNA insertion double mutant of *fancm recq4*.
**Table S1** Primers used in this study.

## Data Availability

Data supporting the findings of this work are available within the paper and its supplementary information files.
