# Supplementary material for: RECQ4 restricts non‐interfering crossover formation to fine‐tune meiotic recombination rates in rice
Source: Plant Biotechnol J. 2025 Jun 15;23(9):3760–9. doi: 10.1111/pbi.70181 (PMC12392947; doi:10.1111/pbi.70181)
Supplement: Supplementary file 1 — Figure S1 Characterization of the mutants. Figure S2 Quantification of DMC1 foci in wild type and the recq4 mutant. Figure S3 Phenotypic comparison between the mus81 mutant and the mus81 recq4 double mutant. Figure S4 Phenotypic comparison between wild type and the recq4 fancm double mutant. Figure S5 Meiotic chromosome behaviours and pollen viability in the T‐DNA insertion double mutant of fancm recq4. Table S1 Primers used in this study. [file PBI-23-3760-s001.docx]

**Supplemental Figures and Table**


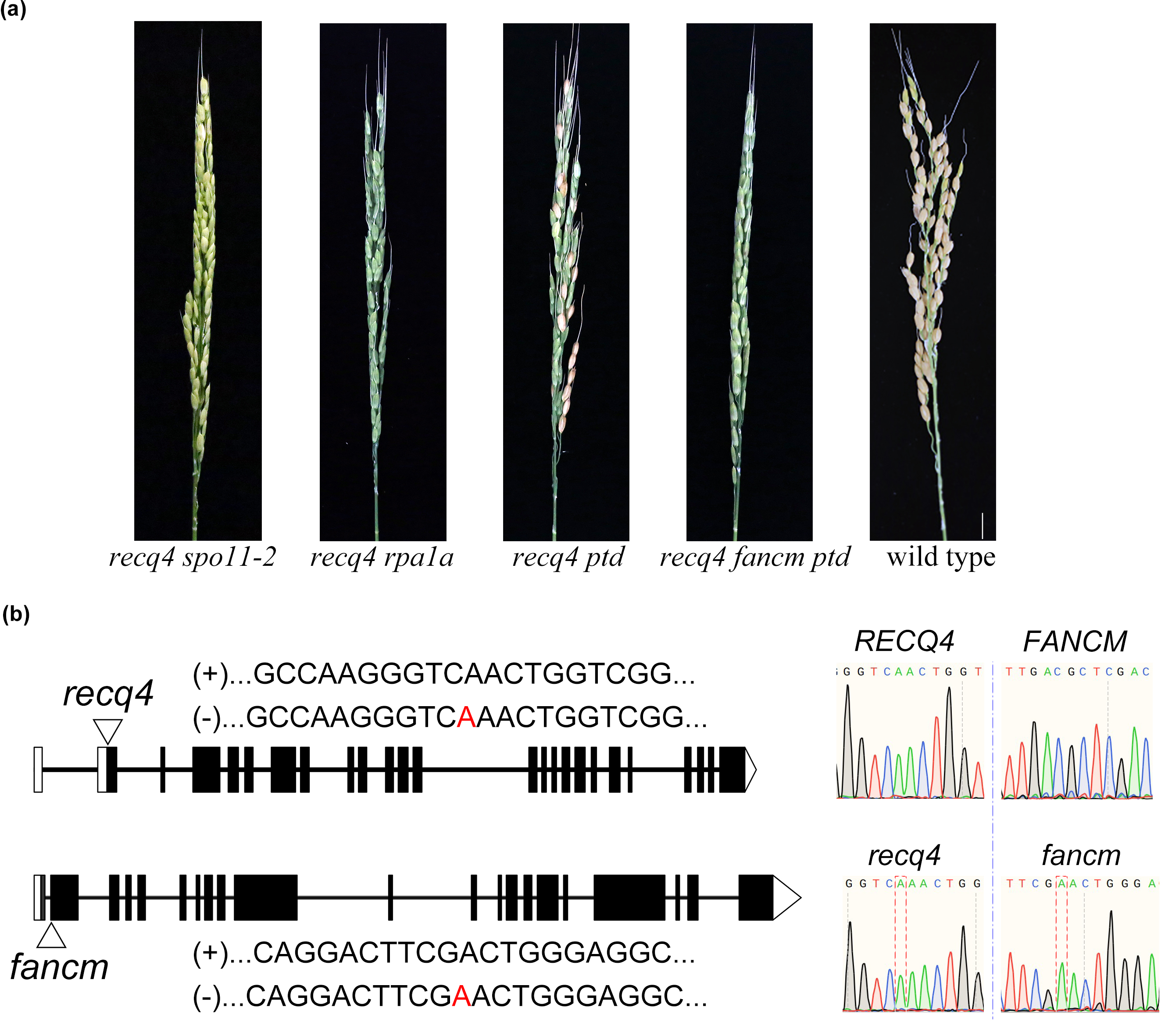


**Figure S1** Characterization of the mutants. (a) The rice panicle with different genotypes. (b) The target sites and mutation types of FANCM and RECQ4.


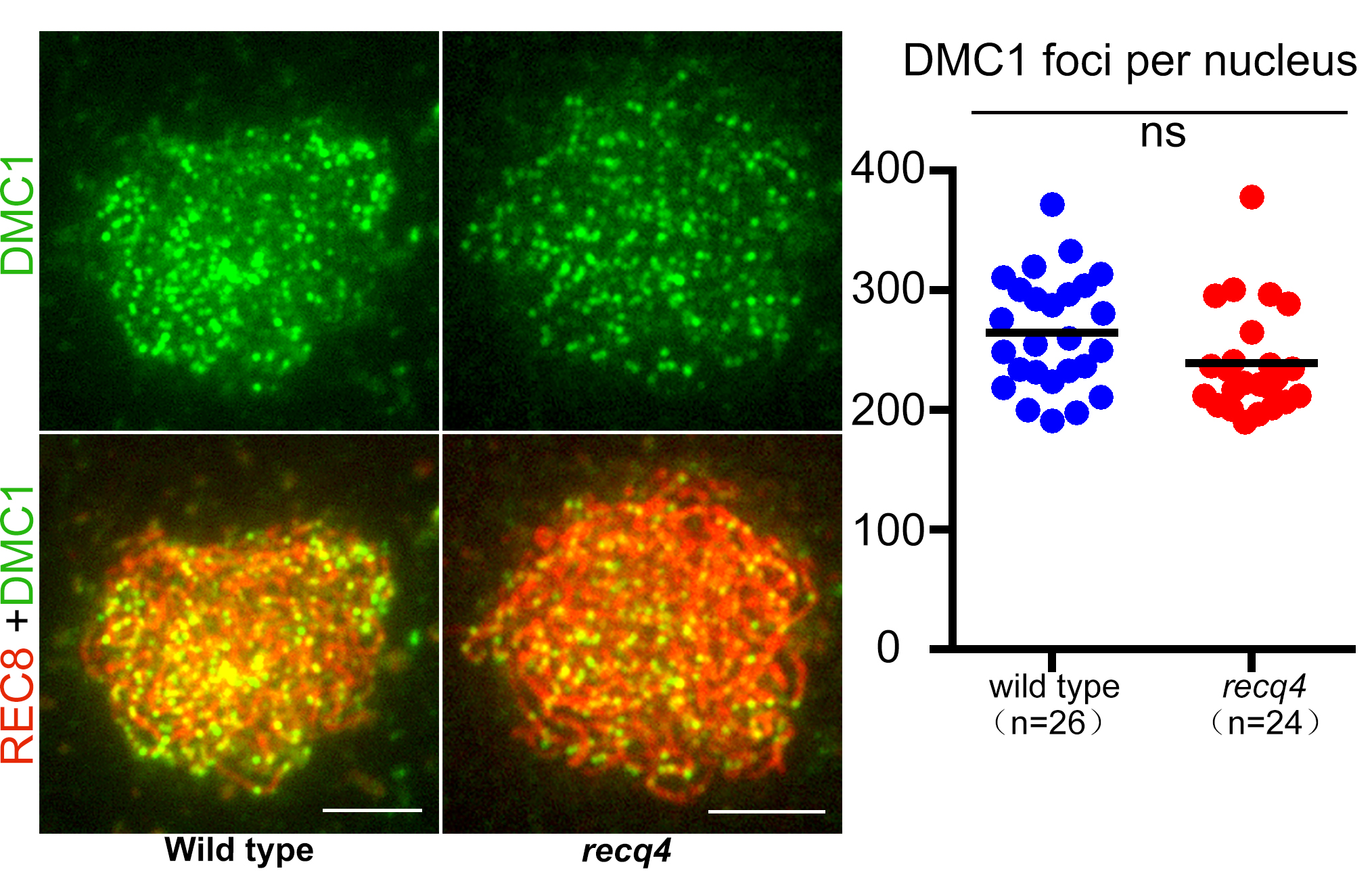


**Figure S2** Quantification of DMC1 foci in wild type and the *recq4* mutant. Statistical analysis showed no significant differences (ns) between wild type and *recq4.* All bars = 5 μm.


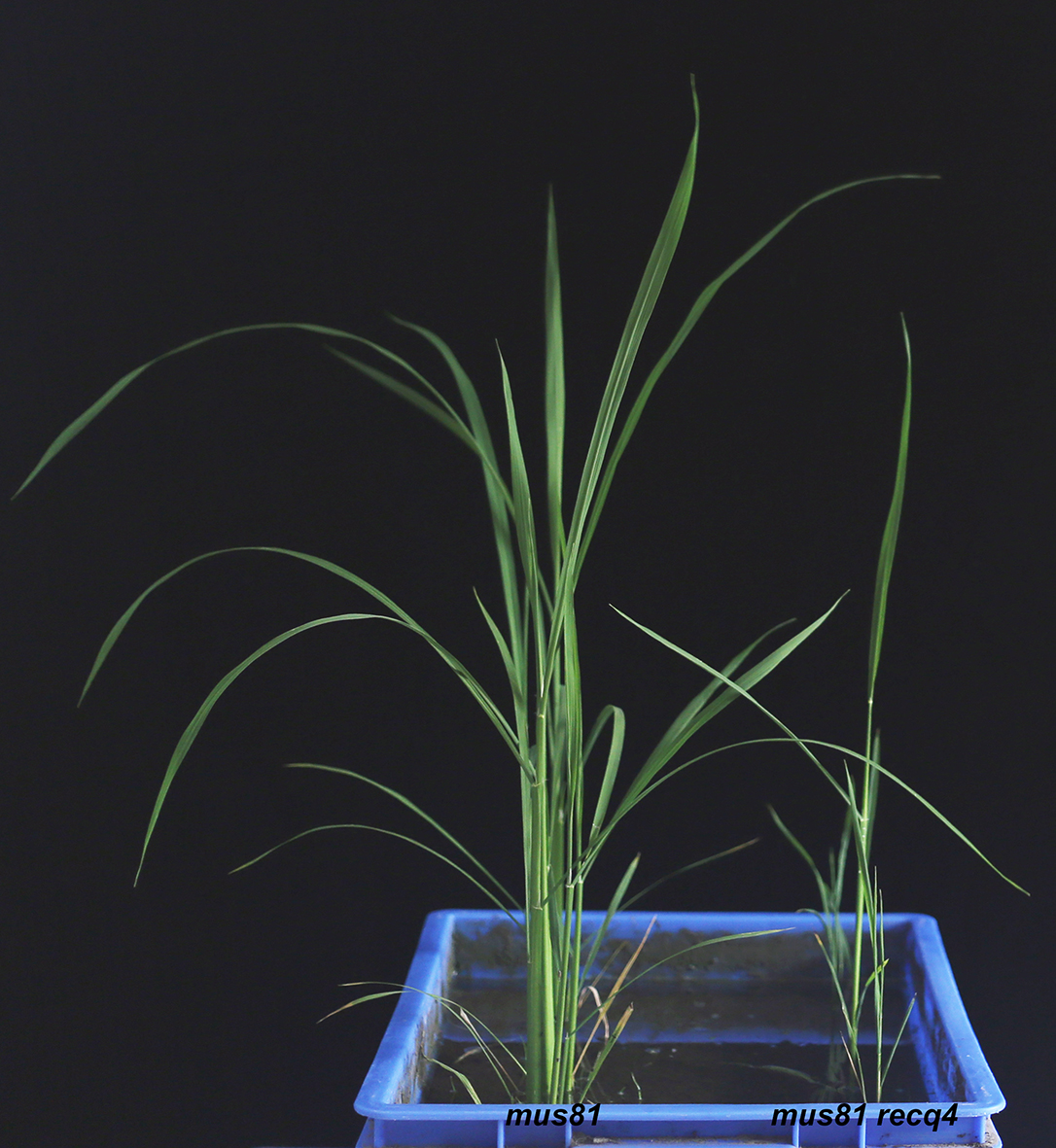


**Figure S3** Phenotypic comparison between the *mus81* mutant and the *mus81 recq4* double mutant.


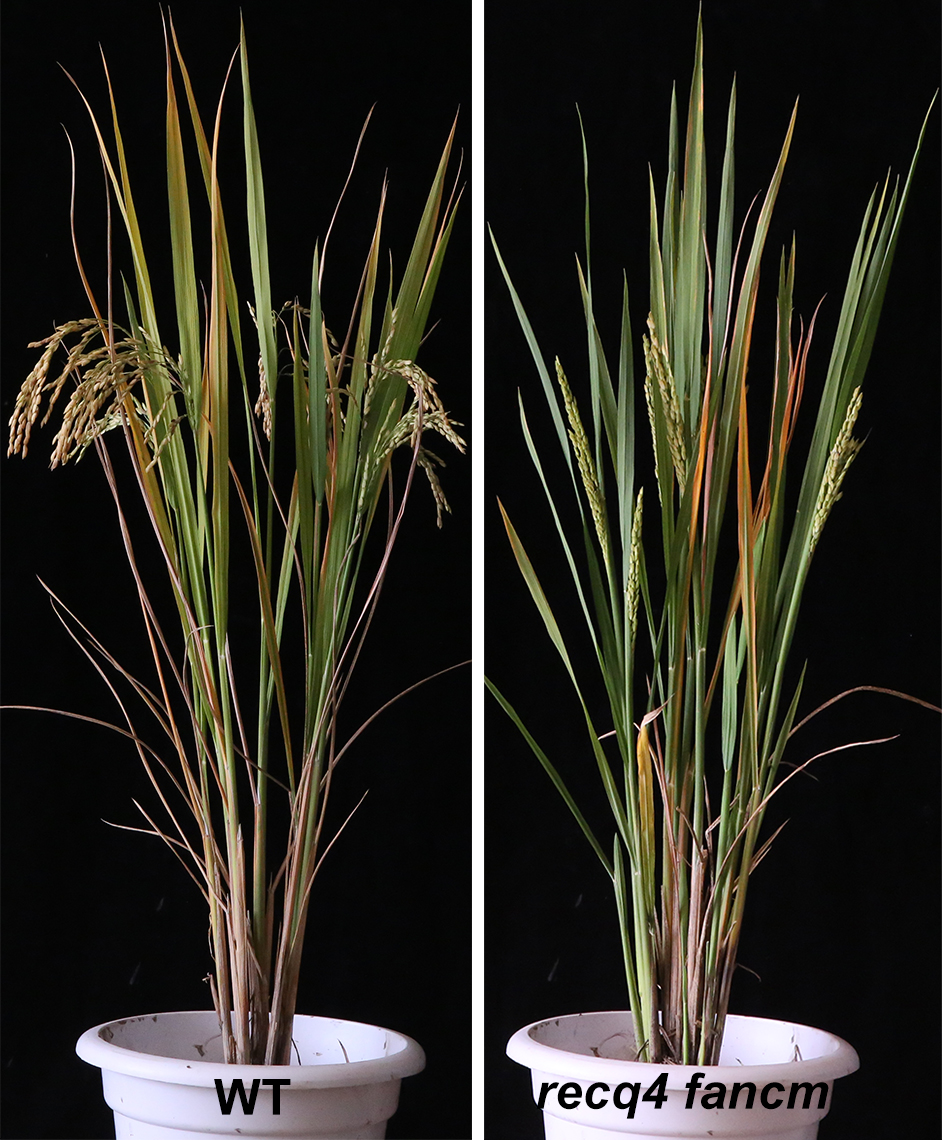


**Figure S4** Phenotypic comparison between wild type (WT) and the *recq4 fancm* double mutant.


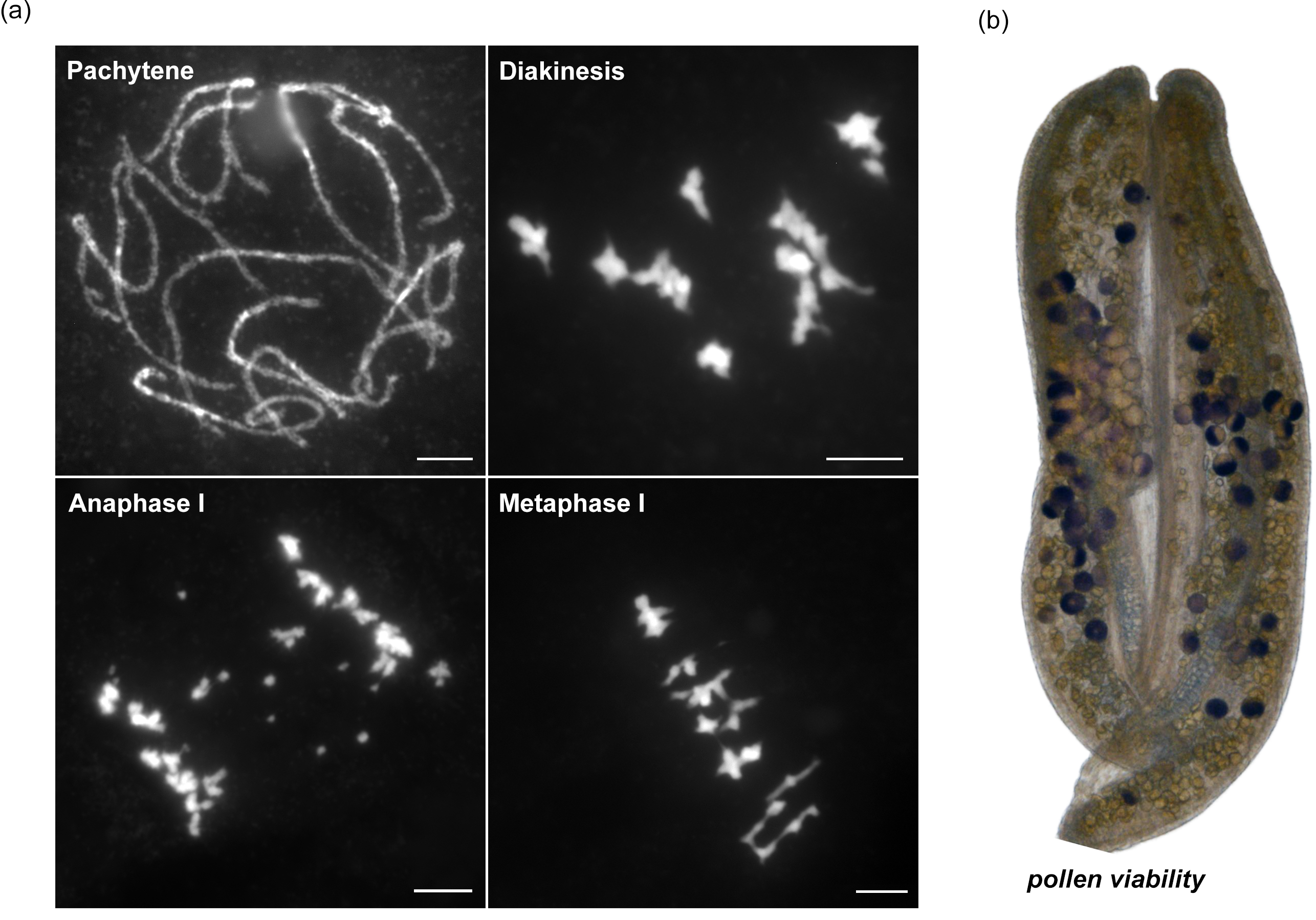


**Figure S5** Meiotic chromosome behaviors and pollen viability in the T-DNA insertion double mutant of *fancm* *recq4.* All bars = 5 μm.

**Table S1** Primers used in this study.

| Primer name | Primer sequence | Description |
| --- | --- | --- |
| *recq4-1*-CAS9-F | GGCATGATAAAGCCAAGGGTCAACTGG | CRISPR/Cas9 Targeting |
| *recq4-1*-CAS9-R | AAACCCAGTTGACCCTTGGCTTTATCA |  |
| *recq4-2*-CAS9-F | GGCAGTTCCCGCAGACGCCAGTCCTGG |  |
| *recq4-2*-CAS9-R | AAACCCAGGACTGGCGTCTGCGGGAAC |  |
| *recq4-3*-CAS9-F | GGCATGACGACTTTGAGGAACGCACGG |  |
| *recq4-3*-CAS9-R | AAACCCGTGCGTTCCTCAAAGTCGTCA |  |
| *fancm-*CAS9-F | GGCAGACTTCGACTGGGAGGCGG |  |
| *fancm-*CAS9-R | AAACCCGCCTCCCAGTCGAAGTC |  |
| *recq4-4-*T-DNA*-*a-F | CCACTTGGGATTAACCGAACATG | T-DNA analysis |
| *recq4-4-*T-DNA*-*a-R | CATTTGCATGATCCGACCAG |  |
| *recq4-4-*T-DNA*-*A-R | CTTGGCTTTATCATGGCAGG |  |
| *fancm-*T-DNA*-*A-F | TATTGCGTGATCGTCGCAGA |  |
| *fancm-*T-DNA*-*a-F | CAGACTGAATGCCCACAGG |  |
| *fancm-*T-DNA*-*Aa-R | AGGAGGCTACTCGGCATTAG |  |
| *zip4*-F | CCTTATTGAGCTCCTCTCTC | Genotyping |
| *zip4*-R | CTCCAGTTGTGAAATGTGCC |  |
| *ptd*-F | TAAGCCCTAAAATTAACTAG |  |
| *ptd*-R | GAAGGTGTTAAAATAGTAAC |  |
| *hei10-F* | CTGCCTGGAAATGACAGC |  |
| *hei10-R* | TAGCTAGCCTCTTGTCCTG |  |
| *mus81-F* | CACCATTCCGGTAAAACCGC |  |
| *mus81-R* | GAAACCTCCCACAAATGGCG |  |
| *rpa1a-F* | AGGAATGAGATCCGAAAGAC |  |
| *rpa1a-R* | AGTCTGCTAATGTAGCGCCG |  |
